# Dietary Knowledge, Attitudes, and Behaviors Related to Obesity and Highly Underweight Among Urban Chinese High School Students: A Cross-Sectional Study

**DOI:** 10.3389/ijph.2024.1606840

**Published:** 2024-08-09

**Authors:** Yujia Huo, Takafumi Monma, Chie Kataoka, Fumi Takeda

**Affiliations:** ^1^ Graduate School of Comprehensive Human Sciences, University of Tsukuba, Tsukuba, Ibaraki, Japan; ^2^ Institute of Health and Sport Sciences, University of Tsukuba, Tsukuba, Ibaraki, Japan

**Keywords:** dietary knowledge, dietary attitudes, dietary behaviors, obesity, highly underweight, high school student, urban China

## Abstract

**Objectives:**

This study aimed to identify dietary knowledge, attitudes, and behaviors related to obesity and highly underweight among urban Chinese high school students.

**Methods:**

Using the data of 403 high school students from a cross-sectional survey in 2022, multinomial logistic regression analysis was conducted with the body mass index as the objective variable (reference: normal weight), dietary knowledge, attitudes, and behaviors as the explanatory variables, adjusted for sex.

**Results:**

Both obesity and highly underweight were most strongly related to incorrect dietary knowledge of desirable types of diets. Additionally, obesity was related to inappropriate dietary attitudes regarding the importance of diet, eating at irregular meal-times, and eating without chewing well, while highly underweight was related to picky eating and not having three meals per day, but not related to attitudes.

**Conclusion:**

The incorrect knowledge of desirable type of diet was related to both obesity and highly underweight, while other risk factors of knowledge, attitudes, and behaviors related to obesity or highly underweight differed respectively. These findings should be useful in examining effective nutrition education for urban Chinese high school students.

## Introduction

In China, the polarization of body types, with increasing rates of obesity and underweight among children and adolescents, has become a public health issue in recent years. Obesity and underweight are defined by a body mass index (BMI) calculated from height and weight. The World Health Organization standard defines a BMI of 30 kg/m^2^ or more as obesity and a BMI of less than 18.5 kg/m^2^ as underweight, but this definition also differs by region or country, as well as by age. For Chinese children and adolescents, BMI is classified as follows: “highly underweight” (male: 16.2 kg/m^2^ or less, female: 16.4 kg/m^2^ or less), “mildly underweight” (male: 16.3–17.3 kg/m^2^, female: 16.5–17.0 kg/m^2^), “normal weight” (male: 17.4–23.2 kg/m^2^, female 17.1–23.5 kg/m^2^), “overweight” (male: 23.3–27 kg/m^2^, female: 23.6–27.0 kg/m^2^), and “obesity” (male: 27.1 kg/m^2^ or more, female: 27.1 kg/m^2^ or more) [1, 2]. Studies using these criteria have reported that individuals aged 6–17 years in China had an overweight/obesity rate of 19.0% in 2020 [3], which has increased, particularly in urban areas with China’s rapid economic growth [4, 5]. Similarly, those aged 6–22 years in China had a mildly/highly underweight rate of 10.2% in 2019 [6]. Moreover, Chinese high school students had a higher rate of underweight than other age groups [7, 8], particularly in urban areas [7].

Furthermore, a survey conducting an international comparison on high school students’ health in 2018 revealed that both the rate of BMI ≥25 kg/m^2^ (17.0%) and BMI <18.5 kg/m^2^ (31.4%) in China were higher than in other Asian countries (e.g., BMI ≥25 kg/m^2^ [5.0%] and BMI <18.5 kg/m^2^ [20.2%] in Japan, and BMI ≥25 kg/m^2^ [13.1%] and BMI <18.5 kg/m^2^ [15.4%] in South Korea) [9]. Children and adolescents with obesity are more likely to develop obesity in adulthood [10, 11], and obesity is a risk factor of the leading cause of death, including heart disease, stroke, diabetes, and various types of cancer [12]. In addition, being highly underweight in childhood and adolescence has adverse health consequences throughout life [13], including stunted growth and hinder maturation, delayed wound healing, hormonal abnormalities, increased susceptibility to infection, and osteoporosis [14]. Therefore, improving both obesity and highly underweight is an urgent issue, and particularly for high school students in urban China.

According to the widely used theoretical model of health education, the KAB model (acronym for Knowledge, Attitudes, and Behaviors) [15], it is vital to possess appropriate knowledge, attitudes, and behaviors to solve health problems. A few studies of Chinese children and adolescents aged 8–18 years and elementary and junior high school students in urban areas have reported characteristics of dietary knowledge, attitudes, and behaviors in individuals with obesity and highly underweight. Regarding dietary knowledge, a study using data from the China Health and Nutrition Survey [16] found that individuals with obesity aged 8–18 years had low dietary knowledge levels [17], but studies on students in elementary and junior high schools [18] and in elementary schools [19] found no association between dietary knowledge and BMI. Regarding dietary attitudes, students with obesity in elementary and junior high schools [18] and those in elementary schools [19] had low dietary attitude levels. Regarding dietary behaviors, students with obesity in elementary schools had low dietary behavior levels [20] and those in elementary and junior high schools had behaviors such as eating at night, picky eating [18] and fast eating [21]. Additionally, highly underweight students in elementary schools had low dietary behavior levels [20].

To the best of our knowledge, most previous studies examined dietary knowledge, attitudes, and behaviors among elementary and junior high school students and analyzed the relationship between those levels and BMI, while no studies have been conducted in high school students. Therefore, this study aimed to identify specific knowledge, attitudes, and behaviors related to obesity and highly underweight among high school students in urban China. This will provide insight into which dietary knowledge, attitudes, and behaviors are crucial in improving obesity or highly underweight among urban Chinese high school students and will be useful in examining effective strategies for nutrition education.

## Methods

### Study Population and Survey

High school students in urban China generally live away from home in boarding houses [22], eat three meals at school cafeterias, and begin to self-determine their diets in the first grade. Therefore, the population of this study was 61,900 first-year high school students in Guangzhou, Guangdong Province, an urban area in China. Based on a required sample size of 402 (calculated with a 5% margin of error, 95% confidence level, and 95% estimated response rate), the survey was administered to 412 randomly selected first-year students (n = 2,160) from four high schools. A survey link was distributed by class teachers to students who provided consent themselves along with their parents and students answered using their personal mobile devices in August 2022. Among the 412 respondents, 403 with complete data (valid response rate: 97.8%) were analyzed.

### Survey Items

The survey items consisted of attributes (sex, height, and weight), dietary knowledge, attitudes, and behaviors, as detailed below. Since there have been no measurements of dietary knowledge, attitudes, and behaviors in high school students, researchers and three high school teachers with extensive health education experience developed those questions, considering content validity appropriate for high school students, based on the “Chinese Physical Education and Health Education Guidelines” [23] and literature.

### Dietary Knowledge

The “Chinese Physical Education and Health Education Guidelines” [23] sets understanding the “Dietary Guidelines for Chinese Residents” [24] as a goal of high school. This guideline includes dietary knowledge of nutrient-dense foods, the function of nutrients in meat, rice, and vegetables, recommended daily fluid intake, desirable diet types, appropriate dietary behaviors, and causes of obesity [24]. Based on them and literature [16, 17], the following 11 questions were developed.

Foods high in carbohydrates (Which of the following foods of equal weight are high in carbohydrates?) was asked to choose from “rice,” “meat,” “butter,” or “cheese.” Foods high in fiber (Which of the following foods of equal weight are high in fiber?) was asked to choose from “bread,” “beans,” “meat,” or “seafood.” Healthy snacks (Which of the following foods qualify as healthy snacks?) was asked to choose from “yogurt or milk,” “sweet bread,” “cup noodles,” or “shortcake.” The functions of meat, etc., (Which of the following functions are performed by meat, fish, eggs, or milk?), the functions of rice, etc., (Which of the following functions are performed by rice, bread, noodles, etc.?), and the functions of vegetables, etc., (Which of the following functions are performed by vegetables, fruits, mushrooms, etc.?) were asked to choose from “to provide energy,” “to build the body,” or “to regulate the body’s condition.” The recommended daily water consumption (Excluding water consumed as food, what is the recommended daily water consumption?) was asked using a four-point scale (“1,200 mL or less,” “1,200–2,000 mL,” “2,000 mL–3,000 mL,” or “3,000 mL or more”). Foods high in calories (Which of the following foods of equal weight has the most calories?) was asked to choose from “bread,” “potato,” “orange,” or “shortcake.” Desirable types of diet (Which of the following diets are desirable?) was asked to choose from a diet “high in protein,” “high in fat,” “free of carbohydrates,” or “contains all nutrients in moderation.” Appropriate dietary behavior (Which of the following dietary behaviors is appropriate?) was asked to choose from “eat snacks frequently,” “eat too fast,” “eat late at night,” or “eat meals at regular times of the day.” Causes of obesity (Which of the following is not a cause of obesity?) was asked to choose from “eat even when full,” “often skip meals,” “often eat thick, fried food,” or “eats three meals per day without skipping a meal.”

### Dietary Attitudes

Since the “Dietary Guidelines for Chinese Residents” [24] does not include dietary attitudes, the following 4 questions were developed with referring a nationwide survey of health education learning evaluation including high school students in Japan [25] and literature [18, 19]: Like of learning about food (Do you like learning about food?), fun of learning about food (Do you think learning about food is fun?), necessity of food learning (Do you think learning about food is necessary?), and importance of diet (Do you think diet is important for a healthy life?) were asked using a five-point rating scale (“strongly agree,” “agree,” “neither agree nor disagree,” “disagree,” or “strongly disagree.”).

### Dietary Behaviors

The “Dietary Guidelines for Chinese Residents” [24] includes dietary behaviors of not eating too fast, eating three meals per day, not picky eating, eating at regular meal-times, reducing consumption of sweet foods, and not eating heavy meals. Based on them and literature [18, 20, 21], the following 8 questions were developed. The frequency of three meals per day (How many days a week do you eat three meals per day?) was asked using a five-point scale (“every day,” “5-6 days,” “3-4 days,” “1-2 days,” or “none”). Eating at night (Do you eat at night?), picky eating (Are you a picky eater?), eating without chewing well (Do you eat without chewing well?), eating at irregular meal-times (Do you eat irregularly?), frequently eating sweet foods (Do you often eat sweet foods?); eating alone (Do you eat alone?), and eating heavy meals (Do you eat heavy meals?) were asked using a five-point scale (“no,” “tend not to,” “neither, ” “tend to,” “yes”).

### Statistical Analysis

BMI was calculated based on height and weight. The BMI classification criteria for children and adolescents in China was determined by the “Chinese BMI Classification Standard for Overweight and Obesity among School-age Children and Adolescents” and the “Chinese BMI Classification Standard for Underweight of School-age Children and Adolescents” [1, 2], as follows: “highly underweight” (male: 16.2 kg/m^2^ or less, female: 16.4 kg/m^2^ or less), “mildly underweight” (male: 16.3–17.3 kg/m^2^, female: 16.5–17.0 kg/m^2^), “normal weight” (male: 17.4–23.2 kg/m2, female 17.1–23.5 kg/m2), “overweight” (male: 23.3–27.0 kg/m^2^, female: 23.6–27.0 kg/m^2^), and “obesity” (male: 27.1 kg/m^2^ or more, female: 27.1 kg/m^2^ or more) [1, 2]. We used these criteria, as they have also been used in previous studies and national surveys in China [26–28].

As for dietary knowledge, answers described below were categorized as “correct:” “rice” for foods high in carbohydrates, “beans” for foods high in fiber, “yogurt or milk” for healthy snack, “to build the body” for function of meat, etc., “to provide energy” for function of rice, etc., “to regulate the body’s condition” for function of vegetables, etc., “1,200–2,000 mL” for recommended daily water consumption, “shortcake” for foods high in calories, “contains all nutrients in moderation” for desirable types of diet, “eat meals at regular times of the day” for appropriate dietary behavior, and “eats three meals a day without skipping a meal” for not causing obesity. Other answers were categorized as “incorrect.”

Answers regarding dietary attitudes were binarized as “agree” (“strongly agree” and “agree”) and “disagree” (“neither agree nor disagree,” “disagree,” “strongly disagree”). Answers regarding dietary behaviors were also binarized as “every day” and “not every day” (“5-6 days,” “3-4 days,” “1-2 days,” or “none”) for the frequency of three meals per day, and as “no” (“no,” “tend not to,” “neither”) and “yes” (“tend to,” “yes”) for other items.

After observing the distribution of respondents’ BMI classification, we analyzed the relationship between BMI groups and dietary knowledge, attitudes, and behaviors using the chi-square test or two-tailed Fisher’s exact test. Three BMI groups (normal weight, highly underweight, and obesity) were selected, and multinomial logistic regression analysis was performed with BMI classification as the objective variable (normal weight: 0); dietary knowledge, attitudes, and behaviors that had significant relationships with BMI in the analysis described above as explanatory variables; and sex as the adjusting variable. The reference categories for this analysis were “correct” for dietary knowledge, “agree” for dietary attitudes, “every day” for frequency of three meals per day, and “no” for other items for dietary behaviors.

IBM SPSS Statistics version 23 (IBM Inc., Armonk, NY, United S) for Windows was used for data aggregation and statistical analyses. All reported values were considered statistically significant at p < 0.05.

## Results

Table 1 shows the distribution of BMI classification of study participants. Individuals with highly underweight comprised 21.3%, those with mildly underweight comprised 9.2%, those with normal weight comprised 50.1%, those with overweight comprised 6.5%, and those with obesity comprised 12.9% of the respondents. Females had a higher rate of highly underweight (26.7%) and normal weight (57.8%) than males, whereas males had a higher rate of overweight (8.8%) and obesity (19.9%) than females.

**TABLE 1:** Distribution of the body mass index classification of respondents, China, 2022.

Total (n)	Highly underweight(n = 86, 21.3%)		Mildly underweight(n = 37, 9.2%)		Normal weight(n = 202, 50.1%)		Overweight(n = 26, 6.5%)		Obesity(n = 52, 12.9%)		*p*-value
	n (%)	AR	n (%)	AR	n (%)	AR	n (%)	AR	n (%)	AR	
Male	36 (16.7)	−2.5*	24 (11.1)	1.4	94 (43.5)	−2.9*	19 (8.8)	2.1*	43 (19.9)	4.5*	<0.001^a^
(216)											
Female	50 (26.7)	2.5*	13 (7.0)	−1.4	108 (57.8)	2.9*	7 (3.7)	−2.1*	9 (4.8)	−4.5*	
(187)											

a Fisher’s exact test.

AR, adjusted residual.

Table 2 shows the relationships between BMI classification and dietary knowledge. Five items, including the function of vegetables, foods high in calories, desirable types of diet, appropriate dietary behavior, and causes of obesity, were significantly related to BMI. For all five items, the obesity group had a significantly lower “correct” response rate: function of vegetables (25.0%), foods high in calories (78.8%), desirable types of diet (38.5%), appropriate dietary behavior (86.5%), and causes of obesity (46.2%). In desirable types of diet, the highly underweight group also had significantly lower “correct” response rate (55.8%). Conversely the normal weight group had a significantly higher “correct” response rate (94.6%).

**TABLE 2:** Relationship between body mass index classification and dietary knowledge, China, 2022.

Items	Total (n = 403)	Highly underweight (n = 86)		Mildly underweight (n = 37)		Normal weight (n = 202)		Overweight (n = 26)		Obesity (n = 52)		*p*-value
	n (%)	n (%)	AR	n (%)	AR	n (%)	AR	n (%)	AR	n (%)	AR	
Foods high in carbohydrates^c^	246 (62.8)	54 (62.8)	0.4	24 (64.9)	0.5	119 (58.9)	−0.9	20 (76.9)	1.7	29 (55.8)	−0.8	0.403^b^
Foods high in fiber^c^	239 (54.7)	47 (54.7)	−1.0	23 (62.2)	0.4	123 (60.9)	0.6	16 (61.5)	0.2	30 (57.7)	−0.3	0.875^a^
Healthy snacks^c^	385 (94.2)	81 (94.2)	−0.7	36 (97.3)	0.5	195 (96.5)	1.0	23 (88.5)	−1.8	50 (96.2)	0.2	0.356^b^
Function of meat, etc.^c^	170 (38.4)	33 (38.4)	−0.8	16 (43.2)	0.1	91 (45.0)	1.2	10 (38.5)	−0.4	20 (38.5)	−0.6	0.799^a^
Function of rice, etc.^c^	286 (62.8)	54 (62.8)	−1.9	26 (70.3)	−0.1	145 (71.8)	0.4	22 (84.6)	1.6	39 (75.0)	0.7	0.245^b^
Function of vegetables, etc.^c^	166 (47.7)	41 (47.7)	1.4	12 (32.4)	−1.1	91 (45.0)	1.6	9 (34.6)	−0.7	13 (25.0)	−2.5*	0.038^b^
Recommended daily water consumption^c^	239 (59.3)	51 (59.3)	0.0	28 (75.7)	2.1	111 (55.0)	−1.8	15 (57.7)	−0.2	34 (65.4)	1.0	0.160^b^
Foods high in calories^c^	367 (94.2)	81 (94.2)	1.1	34 (91.9)	0.2	187 (92.6)	1.1	24 (92.3)	0.2	41 (78.8)	−3.3*	0.046^b^
Desirable types of diet^c^	292 (55.8)	48 (55.8)	−3.9*	18 (48.6)	−3.4*	191 (94.6)	10.0*	15 (57.7)	−1.7	20 (38.5)	−5.9*	<0.001^a^
Appropriate dietary behavior^c^	391 (98.8)	85 (98.8)	1.1	37 (100.0)	1.1	199 (98.5)	1.8	25 (96.2)	−0.3	45 (86.5)	−4.8*	0.002^b^
Causes of obesity^c^	282 (75.6)	65 (75.6)	1.3	34 (91.9)	3.1*	149 (73.8)	1.7	10 (38.5)	−3.6*	24 (46.2)	−4.0*	<0.001^b^

^a^ Chi-square test.

^b^ Fisher’s exact test.

^c^ answer for “correct.”

AR, adjusted residual.

Table 3 shows the relationships between BMI classification and dietary attitudes. Two items—importance of diet and necessity of food learning—were related to BMI. The obesity group had a significantly lower percentage of “agree” responses on the importance of diet (53.8%). Conversely, the highly underweight group had a significantly higher percentage of “agree” responses on the necessity of food learning (89.5%) and the importance of diet (95.3%). The normal weight group had a significantly higher percentage of “agree” responses on the importance of diet (91.1%), whereas they had a lower percentage on the necessity of food learning (75.7%).

**TABLE 3:** Relationship between body mass index classification and dietary attitudes, China, 2022.

Items	Total (n = 403)	Highly underweight (n = 86)		Mildly underweight (n = 37)		Normal weight (n = 202)		Overweight (n = 26)		Obesity (n = 52)		*p*-value
	n (%)	n (%)	AR	n (%)	AR	n (%)	AR	n (%)	AR	n (%)	AR	
Like of learning about food^b^	263 (65.3)	51 (59.3)	−1.3	26 (70.3)	0.7	130 (64.4)	−0.4	18 (69.2)	0.4	38 (73.1)	1.3	0.510^a^
Fun of learning about food^b^	275 (68.2)	52 (60.5)	−1.7	26 (70.3)	0.3	137 (67.8)	−0.2	19 (73.1)	0.5	41 (78.8)	1.8	0.252^a^
Necessity of food learning^b^	321 (79.7)	77 (89.5)	2.6*	33 (89.2)	1.5	153 (75.7)	−2.0*	20 (76.9)	−0.4	38 (73.1)	−1.3	0.022^a^
Importance of diet^b^	347 (86.1)	82 (95.3)	2.8*	37 (100.0)	2.6*	184 (91.1)	2.9*	16 (61.5)	−3.7*	28 (53.8)	−7.2*	<0.001^a^

^a^ Fisher’s exact test.

^b^ answer for “agree”.

AR, adjusted residual.

Table 4 shows the relationships between BMI classification and dietary behaviors. Four items—frequency of three meals per day, picky eating, eating without chewing well, and eating at irregular meal-times—were related to BMI. The obesity group had significantly higher percentage of “every day” responses on the frequency of three meals per day (80.8%), “yes” on eating without chewing well (71.2%), and eating at irregular meal-times (73.1%), whereas they had lower rates of “yes” responses to picky eating (30.8%). The highly underweight group had a significantly higher percentage of “yes” responses to picky eating (80.2%), whereas significantly lower rates of “every day” responses to the frequency of three meals per day (46.5%) and of “yes” on eating without chewing well (8.1%).

**TABLE 4:** Relationship between body mass index classification and dietary behaviors, China, 2022.

Items	Total (n = 403)	Highly underweight (n = 86)		Mildly underweight (n = 37)		Normal weight (n = 202)		Overweight (n = 26)		Obesity (n = 52)		*p*-value
	n (%)	n (%)	AR	n (%)	AR	n (%)	AR	n (%)	AR	n (%)	AR	
Frequency of three meals per day^c^	259 (64.3)	40 (46.5)	−3.9*	17 (45.9)	−2.4*	140 (69.3)	2.1*	20 (76.9)	1.4	42 (80.8)	2.7*	<0.001^a^
Eating at night^d^	159 (39.5)	33 (38.4)	−0.2	17 (45.9)	0.8	76 (37.6)	−0.8	10 (38.5)	−0.1	23 (44.2)	0.8	0.829^b^
Picky eating^d^	204 (50.6)	69 (80.2)	6.2*	27 (73.0)	2.9*	85 (42.1)	−3.4*	7 (26.9)	−2.5*	16 (30.8)	−3.1*	<0.001^a^
Eating without chewing well^d^	104 (25.8)	7 (8.1)	−4.2*	7 (18.9)	−1.0	37 (18.3)	−3.4*	16 (61.5)	4.3*	37 (71.2)	8.0*	<0.001^a^
Eating at irregular meal-times^d^	139 (34.5)	27 (31.4)	−0.7	9 (24.3)	−1.4	46 (22.8)	−5.0*	19 (73.1)	4.3*	38 (73.1)	6.3*	<0.001^a^
Frequently eating sweet foods^d^	203 (50.4)	49 (57.0)	1.4	20 (54.1)	0.5	96 (47.5)	−1.1	15 (57.7)	0.8	23 (44.2)	−0.9	0.448^b^
Eating alone^d^	141 (35.0)	29 (33.7)	−0.3	13 (35.1)	0.0	71 (35.1)	0.1	10 (38.5)	0.4	18 (34.6)	−0.1	0.995^b^
Eating heavy meals^d^	169 (41.9)	34 (39.5)	−0.5	14 (37.8)	−0.5	85 (42.1)	0.1	12 (46.2)	0.5	24 (46.2)	0.7	0.905^b^

^a^ Fisher’s exact test.

^b^ Chi-square test.

^c^ answer for “every day”.

^d^ answer for “yes”.

AR, adjusted residual.

Table 5 shows the results of the multinomial logistic regression analysis with BMI classification (reference category: normal weight) as the objective variable; dietary knowledge, attitudes, and behaviors as explanatory variables; and sex as the adjusting variable. The highly underweight group had higher odds ratios (ORs) for incorrect dietary knowledge about desirable types of diet (OR 13.88), dietary attitude disregarding the necessity of food learning (OR 0.37), and inappropriate dietary behaviors of not eating three meals per day (OR 2.59), picky eating (OR 5.59), and eating without chewing well (OR 0.40) than did the normal weight group. On the other hand, the obesity group had higher ORs for incorrect dietary knowledge about the function of vegetables, etc. (OR 2.23), foods high in calories (OR 2.84), desirable types of diet (OR 26.32), appropriate dietary behavior (OR 8.00), and causes of obesity (OR 2.59), inappropriate dietary attitude about disregarding the importance of diet (OR 8.71), and inappropriate dietary behaviors, such as eating without chewing well (OR 9.95) and eating at irregular meal-times (OR 12.56) than did the normal weight group.

**TABLE 5:** Multinomial logistic regression analysis for body mass index classification (highly underweight and obesity), China, 2022.

		Highly underweight			Obesity		
		OR	95%CI	*p*-value	OR	95%CI	*p*-value
Dietary knowledge							
Function of vegetables, etc.	Incorrect (Ref. correct)	0.91	0.55–1.51	0.719	2.23	1.10–4.51	0.026
Foods high in calories	Incorrect (Ref. correct)	0.79	0.28–2.24	0.654	2.84	1.17–6.89	0.021
Desirable types of diet	Incorrect (Ref. correct)	13.88	6.60–29.21	<0.001	26.32	11.24–61.61	<0.001
Appropriate dietary behavior	Incorrect (Ref. correct)	0.81	0.08–7.94	0.857	8.00	1.88–34.04	0.005
Causes of obesity	Incorrect (Ref. correct)	0.94	0.52–1.70	0.834	2.59	1.35–4.97	0.004
Dietary attitudes							
Necessity of food learning	Disagree (Ref. agree)	0.37	0.17–0.78	0.010	1.16	0.56–2.37	0.695
Importance of diet	Disagree (Ref. agree)	0.50	0.16–1.52	0.222	8.71	4.01–18.95	<0.001
Dietary behaviors							
Frequency of three meals per day	Not every day (Ref. every day)	2.59	1.53–4.38	<0.001	0.70	0.32–1.53	0.374
Picky eating	Yes (Ref.no)	5.59	3.06–10.22	<0.001	0.73	0.37–1.44	0.369
Eating without chewing well	Yes (Ref.no)	0.40	0.17–0.94	0.035	9.95	4.84–20.47	<0.001
Eating at irregular meal-times	Yes (Ref.no)	1.52	0.86–2.68	0.151	12.56	5.92–26.62	<0.001

OR, odds ratios; CI, confidence interval.

Multinomial logistic regression analysis was conducted with BMI classification (reference category: normal weight) as the objective variable; dietary knowledge, attitudes, and behaviors as explanatory variables; and sex as the adjusting variable.

## Discussion

This study provided insight into the dietary knowledge, attitudes, and behaviors related to obesity and highly underweight among high school students in urban China.

Among the study respondents, the rates of the obesity/overweight and mildly/highly underweight groups were 19.4% and 30.5%, respectively. This was similar to the results reported by the China Youth and Children Research Center on high school students in urban China (17.0% obesity/overweight, 31.4% mildly underweight/highly underweight) [9]. Additionally, among the respondents, the rate of obesity was higher in males, while that of highly underweight was higher in females. This was consistent with previous findings on high school students in other urban areas in China [22, 29]. Thus, the distribution of BMI among the participants of this study might be generalizable among high school students in urban China.

Among our respondents, the obesity group tended to have incorrect dietary knowledge (e.g., about the function of vegetables, foods high in calories, desirable types of diet, appropriate dietary behavior, and causes of obesity) and inappropriate attitudes regarding the importance of diet. In terms of dietary behaviors, they tended to exhibit undesirable behaviors, such as eating without chewing well and eating at irregular meal-times, whereas they also exhibited desirable behaviors, such as eating three meals per day and not picky eating. These findings supported low dietary knowledge levels of individuals with obesity aged 8–18 years [17] and low dietary attitude levels of students with obesity in elementary and junior high school [18, 19], whereas did not support no relationship between dietary knowledge and BMI [18, 19] and inappropriate behaviors of picky eating [18] in students with obesity in elementary and junior high school. Second, the highly underweight group tended to have incorrect dietary knowledge about the desirable types of diet and inappropriate dietary behaviors, such as not eating three meals per day and being picky eaters, whereas they tended to have appropriate attitudes about the necessity of food learning and the importance of diet. Our findings were inconsistent with previous ones of no relationship between dietary knowledge and BMI [18, 19] and of inappropriate attitudes of highly underweight [20] in elementary and junior high school students. Thus, it is possible that the dietary habits of high school students with obesity and highly underweight have unique characteristics different from those of elementary and junior high school students, since it was known that students’ dietary knowledge [18], attitudes [25], and behaviors [30] levels differed by school grades.

Multinomial logistic regression analysis with adjustment for sex showed that the strongest risk factor for obesity was inaccurate dietary knowledge of desirable types of diet (OR 26.32), followed by inappropriate dietary behaviors, such as eating at irregular meal-times (OR 12.56) and eating without chewing well (OR 9.95), and having an inappropriate attitude toward the importance of diet (OR 8.71). Additionally, inaccurate knowledge of appropriate dietary behavior (OR 8.00), food high in calories (OR 2.84), causes of obesity (OR 2.59), and the function of vegetables (OR 2.23) were risk factors for obesity. Our findings were consistent with those of previous studies that reported a relationship between obesity and inappropriate dietary behaviors, such as eating without chewing well [21] in Chinese children and adolescents, but were inconsistent with those of studies in elementary school students, which reported that the obesity group had inappropriate dietary behaviors, such as picky eating [18]. Second, the strongest risk factor for highly underweight was incorrect dietary knowledge about the desirable types of diet (OR 13.88), followed by inappropriate dietary behaviors of picky eating (OR 5.59) and not having three meals per day (OR 2.59). On the other hand, individuals with highly underweight had appropriate dietary attitudes regarding the necessity of food learning (OR 0.37) and appropriate dietary behavior of chewing well when eating (OR 0.40).

These findings suggest that incorrect dietary knowledge of desirable types of diet was the strongest risk factor for both obesity (OR 26.32) and highly underweight (OR 13.88). Therefore, acquiring proper knowledge that a diet that contains all nutrients in moderation is desirable may be effective in improving both obesity and highly underweight among high school students in urban China. Second, obesity had various risk factors, such as undesirable dietary behaviors of eating at irregular meal-times (OR 12.56) and eating without chewing well (OR 9.95), and inappropriate dietary attitudes regarding the importance of diet (OR 8.71), as well as incorrect dietary knowledge of appropriate dietary behavior (OR 8.00), food high in calories (OR 2.84), causes of obesity (OR 2.59), and the function of vegetables (OR 2.23). Therefore, it is necessary for obese students to have nutrition education about eating regular meals, chewing well when eating, and appropriate cognition of the importance of diet. Third, the risk factors for highly underweight, besides the aforementioned incorrect dietary knowledge about desirable types of diet, were only two inappropriate dietary behaviors: picky eating (OR 5.59) and not having three meals per day (OR 2.59). In urban China, high school students generally eat three meals a day in the self-service school cafeteria, where students must choose their own main and side dishes from various menus each day, not a set menu [31]. Therefore, it is important to examine nutrition education that improves the aforementioned knowledge, attitudes, and behaviors that are risk factors for obesity and highly underweight.

This study had several limitations. First, as this was a cross-sectional study and the dietary instruments did not include temporality information in the questions, we could not clarify the causal relationships between dietary knowledge, attitudes, behaviors, and BMI. Future longitudinal studies are required to verify these causal relationships. Second, as the data were collected using self-reported questionnaires, reporting bias cannot be ruled out. Further uniform studies using objective measurements of height, weight, and dietary habits should be conducted. Third, the study population was limited to high school students in one urban area; therefore, further studies should be conducted in other urban areas. Fourth, the instruments of dietary knowledge, attitudes and behaviors in this study were developed with considering content validity appropriate for high school students, but not verified objective validity, thus it is necessary to verify the validation of instruments in future research. Lastly, since this study examined only theoretical aspects of KAB model, but not other aspects of lifestyle habits of physical activity, weight-loss, and sleep, health problems including eating disorder, and socio-economic and genetic factors, further examination considering multiple factors is need.

Despite these limitations, this is the first study to clarify dietary knowledge, attitudes, and behaviors related to obesity and highly underweight among high school students in urban China. The key strength of this study lies in its elucidation of the following: Since risk factors for dietary habits were stronger and more varied in individuals with obesity than in those with highly underweight, which differed between these groups (besides the incorrect dietary knowledge of desirable types of diet), it is necessary to consider each characteristic to identify measures for improving obesity and highly underweight. In this context, nutrition education and school cafeteria menus should be examined.

### Conclusions

This study identified dietary knowledge, attitudes, and behaviors related to obesity and highly underweight among high school students in urban China. The results showed that a lack of knowledge of desirable types of diet was the strongest risk factor for both obesity and highly underweight. In addition, inappropriate dietary behaviors, such as eating at irregular meal-times, eating without chewing well, inappropriate dietary attitudes regarding the importance of diet, and other inaccurate dietary knowledge were risk factors for obesity, whereas inappropriate dietary behaviors of picky eating and not having three meals per day were risk factors for highly underweight. These findings will be useful in future nutrition education to prevent and improve obesity and highly underweight among high school students in urban China.
